# Knowledge graph prediction of unknown adverse drug reactions and validation in electronic health records

**DOI:** 10.1038/s41598-017-16674-x

**Published:** 2017-11-27

**Authors:** Daniel M. Bean, Honghan Wu, Ehtesham Iqbal, Olubanke Dzahini, Zina M. Ibrahim, Matthew Broadbent, Robert Stewart, Richard J. B. Dobson

**Affiliations:** 1 Department of Biostatistics and Health Informatics, Institute of Psychiatry Psychology and Neuroscience, King’s College London, 16 De Crespigny Park, London, SE5 8AF United Kingdom; 2 South London and Maudsley NHS Foundation Trust, Denmark Hill, London, SE5 8AZ United Kingdom; 3 Institute of Pharmaceutical Science, King’s College, London, 5th Floor, Franklin-Wilkins Building, 150 Stamford Street, London, SE1 9NH United Kingdom; 4 Institute of Psychiatry, Psychology and Neuroscience, King’s College London, 16 De Crespigny Park, London, SE5 8AF United Kingdom; 5 Farr Institute of Health Informatics Research, UCL Institute of Health Informatics, University College London, London, WC1E 6BT United Kingdom

## Abstract

Unknown adverse reactions to drugs available on the market present a significant health risk and limit accurate judgement of the cost/benefit trade-off for medications. Machine learning has the potential to predict unknown adverse reactions from current knowledge. We constructed a knowledge graph containing four types of node: drugs, protein targets, indications and adverse reactions. Using this graph, we developed a machine learning algorithm based on a simple enrichment test and first demonstrated this method performs extremely well at classifying known causes of adverse reactions (AUC 0.92). A cross validation scheme in which 10% of drug-adverse reaction edges were systematically deleted per fold showed that the method correctly predicts 68% of the deleted edges on average. Next, a subset of adverse reactions that could be reliably detected in anonymised electronic health records from South London and Maudsley NHS Foundation Trust were used to validate predictions from the model that are not currently known in public databases. High-confidence predictions were validated in electronic records significantly more frequently than random models, and outperformed standard methods (logistic regression, decision trees and support vector machines). This approach has the potential to improve patient safety by predicting adverse reactions that were not observed during randomised trials.

## Introduction

Hospital admissions resulting from adverse drug reactions (ADRs) have been projected to cost the National Health Service £466m^1^. A meta-analysis of US hospitals estimated the incidence of serious ADRs at 6.7%^2^. ADRs are therefore a significant risk to patient health, treatment compliance and healthcare costs. ADRs are also a key factor in the cost-benefit analysis of pharmacological treatments. This analysis is critical to the decision making process for drug licensing and prescription. Although ADRs are monitored during clinical trials, practical limitations on sample size and study population mean not all ADRs of a drug will be detected before it is approved for use. Ongoing pharmacovigilance and monitoring of drugs for post-marketing side effects is therefore essential. Spontaneous reports of ADRs are sent to regulatory bodies such as the US Food and Drug Administration (via the FDA Adverse Event Reporting System, FAERS^3^), the World Health Organisation (via VigiBase^4^), the UK Medicines and Healthcare Products Regulatory Agency (via the yellow card scheme^5^) or the European Medicines Agency (via EudraVigilance^6^). These reports may eventually end up in drug product inserts, or could result in a drug being withdrawn from the market. Unfortunately, post-marketing surveillance is limited by under-reporting of ADRs due to time constraints, limited training in reporting procedures and the low perceived impact of an individual report, amongst other factors^7^.

Until enough reports emerge for a previously unknown ADR to be recognised as such, these unknown ADRs pose a risk to patients, limit the accuracy of cost-benefit analysis and lead to unexpected healthcare costs. The ability to predict ADRs is therefore highly desirable, and has been the subject of numerous previous studies (reviewed in^8,9^). *In silico* prediction of the safety profile of a candidate molecule has the advantage of extremely high throughput and is increasingly a part of the lead optimisation pipeline in drug discovery^10^. Similar methods can be applied to marketed drugs, and may benefit from the increased data available on the drug. The aims of the research reported in this paper were to predict additional (unknown) ADRs for drugs currently in use, and to verify those predictions using information extracted from anonymised electronic health records (EHRs).

“Knowledge graphs” represent facts as edges between nodes that represent entities (e.g. people, drugs) or concepts (e.g. actor, migraine). Regardless of the specific technology used to create them, representing facts as a graph allows both highly efficient querying and automated reasoning. We constructed a knowledge graph containing publicly available data on drugs, their target proteins, clinical indications and known ADRs. In the context of this graph, unknown ADRs for drugs are missing edges between drugs nodes and ADR nodes. This graph is the input to our prediction algorithm, which predicts unknown ADRs by inferring missing edges in the graph.

Edges may be absent from the graph for three main reasons: 1) The drug does not cause the ADR, 2) The drug can cause the ADR and this fact is known but missing from the source database, 3) The drug can cause the ADR but is not yet known to. The aim of the algorithm presented in this paper is to place new edges in the graph that fall into categories 2 or 3. Importantly, correct predictions in these two classes are equivalent in terms of validating the prediction algorithm, even though the category 2 edges (known but missing from the graph) are known elsewhere. The correct prediction of edges in category 2 does not directly contribute to patient care, but as these databases are widely used for research purposes it is valuable to detect missing information.

## Predicting unknown ADRs

ADR prediction has been the subject of numerous previous publications, which have been reviewed thoroughly^8,9^. Existing approaches can be subdivided into two key objectives. Firstly, to predict ADRs for a lead compound before marketing, and secondly, to make predictions that add new ADRs to the existing profile. The work presented here falls into the relatively uncommon category of predicting new ADRs for drugs in the post-marketing period. In this same category of study, Cami *et al*.^11^ trained a logistic regression classifier using structural properties of the drug-ADR network together with chemical and taxonomic properties of drugs as features to predict unknown ADRs for marketed drugs. The authors tested the predictive performance of their model in a simulated prospective framework using snapshots from a commercial database of spontaneous ADR reports; the best model achieved an AUROC of 0.87 with a sensitivity of 0.42. We use the same classification method as one of the benchmarks for the performance of our algorithm. Rahmani *et al*.^12^ predicted unknown ADRs by applying a random walk algorithm to a network with drug and ADR nodes, where drug-ADR edges represent known ADRs and drug-drug edges indicate drug target similarity, but did not validate new ADRs in any real-world clinical data. Bresso *et al*.^13^ constructed a database of drug, ADR and target knowledge and used decision trees and inductive logic programming to predict ADR profiles (rather than individual ADRs) and validated predicted ADRs using FAERS^3^.

These previous studies demonstrate the ability of existing machine learning algorithms to predict new ADRs for marketed drugs, but are limited in terms of validation. Spontaneous reports are one of the foundations of post-marketing pharmacovigilance and are widely used as validation data in ADR prediction, however these databases depend on reports being submitted, and further on the accuracy of those reports. Under-reporting of ADRs^7^ significantly limits the use of such databases both for ADR detection and as a validation set for a prediction task. To address this issue, electronic health records (EHRs) can potentially be analysed to detect ADRs^8^, removing the dependency on reporting. Data mining from EHRs has been used to detect novel ADRs^14^, or combined with spontaneous reports to increase confidence in detected drug-ADR signals^15^. Reliably extracting ADR mentions from the free text of EHRs is challenging – a single concept may be described in several different specific ways (e.g. synonyms, acronyms or shorthand), or may be mentioned in a historical context. We therefore focus on a subset of ADRs for which the NLP pipeline is validated^16^.

## Basis for the prediction algorithm

**Figure 1:**
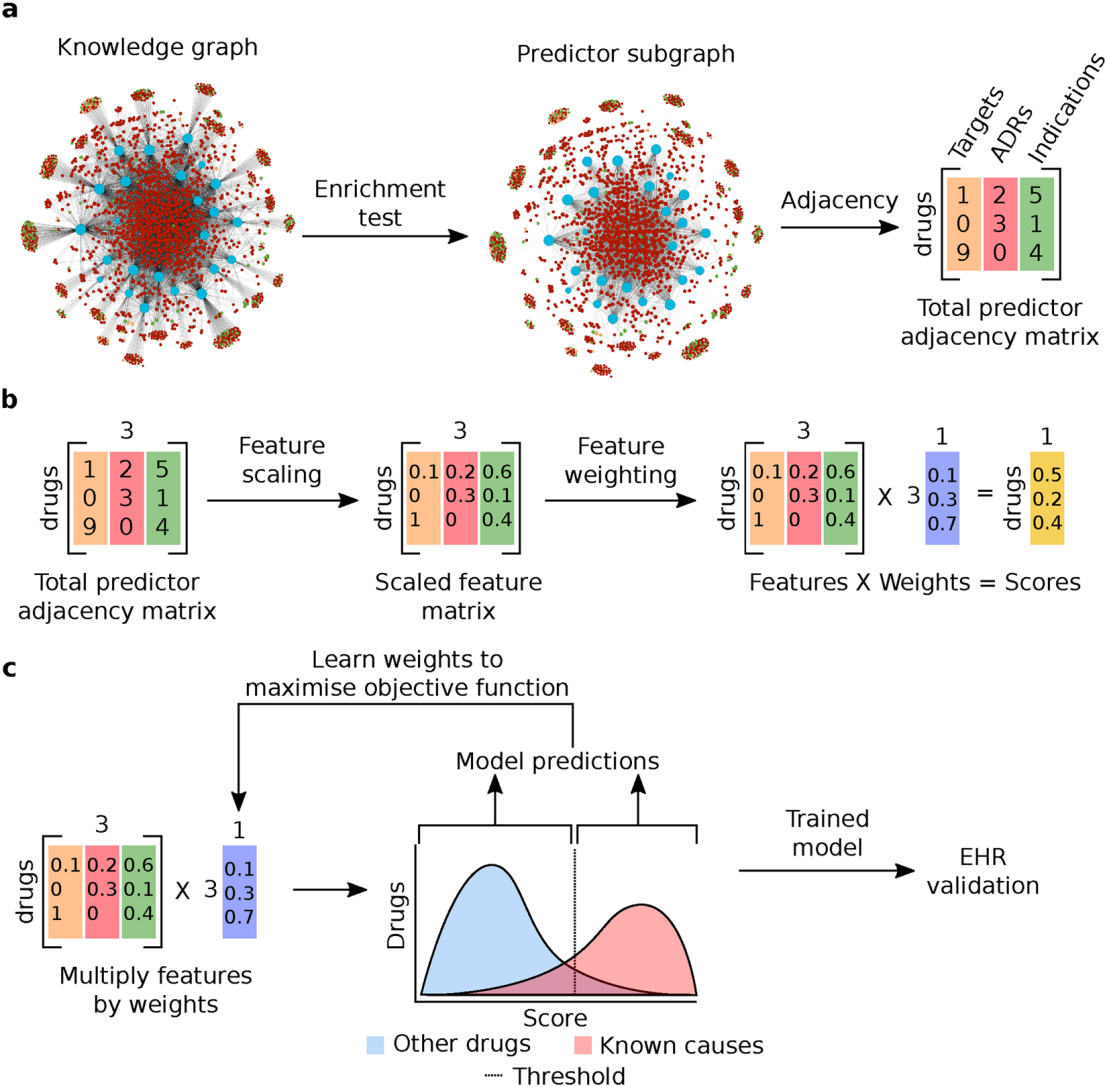
Overview of the prediction algorithm. (**a**) Starting from a knowledge graph containing all publicly available information on the ADR being predicted, an enrichment test is used to identify predictive features of the drugs known to cause the ADR. The total adjacency of every drug with all predictors of each type (the columns of the matrix) is calculated from the graph. Blue nodes are drugs, red nodes are ADRs, orange nodes are targets, green nodes are indications. (**b**) The features (adjacency matrix from (**a**)) are scaled and weighted to produce a final score for every drug. (**c**) The optimum weight vector from (**b**) is learned from the knowledge graph to maximize an objective function. The predictions of this optimized model are tested in EHRs.

The workflow of the prediction algorithm is shown in Fig. 1. Intuitively, the drugs that cause a given ADR should have certain features in common that are related to the mechanism(s) by which they cause the ADR, such as protein targets, transporters or chemical features. One way to identify these features is by a simple enrichment test^17,18^. The enrichment test is a simplistic way to mimic the human reasoning process. For example, for any given ADR of interest the known causes are likely to also cause nausea, but we wouldn’t predict that therefore any drug that can cause nausea could be a cause of our ADR of interest because we know most drugs can cause nausea. In other words nausea is not a specific feature of the known causes of the ADR and we would ignore it. The result is achieved in our method by testing for the enrichment of each feature (e.g. also causing nausea or targeting a specific protein) for all the known causes of an ADR of interest vs all other drugs. Features that are found to be significantly enriched for the known causes of an ADR are used in the predictive model, other features are not included.

This set of features can be thought of as a “meta-drug” with only the enriched features of the known causes of an ADR. Any drug can now be scored for its similarity to this profile, and we expect the known causes of this ADR to score relatively highly. Any drug that is not currently known to cause the ADR but that also has a high similarity to the enriched “meta-drug” profile is predicted to be a potential new cause. This process is repeated for every ADR to generate new predictions.

The features produced from the knowledge graph (in conjunction with the enrichment test) can be used for classification by any standard machine learning algorithm such as logistic regression (LR), decision trees (DT) and support vector machines (SVM). These three methods are used as a benchmark for the method presented here. Our method is most similar to LR, indeed the hypothesis functions can be stated equivalently. The significant difference between our method and LR is the objective function used to optimise the feature weights. In LR the weight vector is selected to maximising the (log) likelihood, whereas our method optimises Youden’s J statistic (see Methods).

The performance of the prediction algorithm on the constructed knowledge graph was assessed in 3 ways: 1) ability to correctly classify the known causes of each ADR, 2) ability to predict (replace) edges deleted from the graph, 3) ability to predict ADRs not present in the graph but observed in EHRs. The presented prediction algorithm performed well across all tests, indicating that automated reasoning from knowledge graphs representing publicly available knowledge can be used to accurately predict unknown ADRs that have been observed in clinical practice. Filling in the blanks in our knowledge of ADRs would potentially reduce risks to patients and associated healthcare costs.

## Results

### Construction of the drug knowledge graph

Public data on drug targets, indications and ADRs were retrieved and integrated to create the drug knowledge graph. Only marketed drugs with at least one edge of each type were retained in the graph. The final graph contains 70,382 edges (clinical indication, protein target, adverse reactions) for 524 drugs (Table 1). The distributions of numbers of known causes per ADR and known ADRs per drug were both highly skewed (Supplementary Figure S1), with a median of 3 known causes per ADR and 85 known ADRs per drug. The most common ADR was nausea, which is a known reaction to 88% of drugs in the graph, followed by headache (86%) and rash (81%). 32% of all ADR nodes in the graph have only a single known cause, for example Acrodynia (discolouration and pain of hands and feet, a rare side effect of Riluzole).

**Table 1:** Size of the drug knowledge graph. Raw data was filtered to retain only marketed drugs with at least one known ADR, target and indication.

Network	Drug nodes	Other nodes	Total nodes	Edges
Drug-ADR	524	3144	3668	62380
Drug-Target	524	736	1260	2610
Drug-Indication	524	1424	1948	5392
Total	524	5304	5828	70382

### Model performance in a simulated prediction task

The goal of the prediction algorithm is to use knowledge about drugs known to cause an ADR to predict new causes, which is equivalent to adding edges in the knowledge graph. To simulate this task, a proportion of the existing drug-ADR edges for each ADR are deleted from the graph before training a predictive model. The performance of the model is evaluated using the proportion of the deleted edges that it correctly placed. Importantly, and unlike a standard k-fold cross validation, the test set of drugs is included in the training data, but as true negatives. This is an exact simulation of the intended use-case: adding new edges to nodes already present in the graph. This procedure was performed over 10 folds for each ADR in the graph (meaning in each fold 10% of the “known cause” edges from this ADR to drug nodes are deleted). Deleted edges are replaced before beginning the next fold.

**Figure 2:**
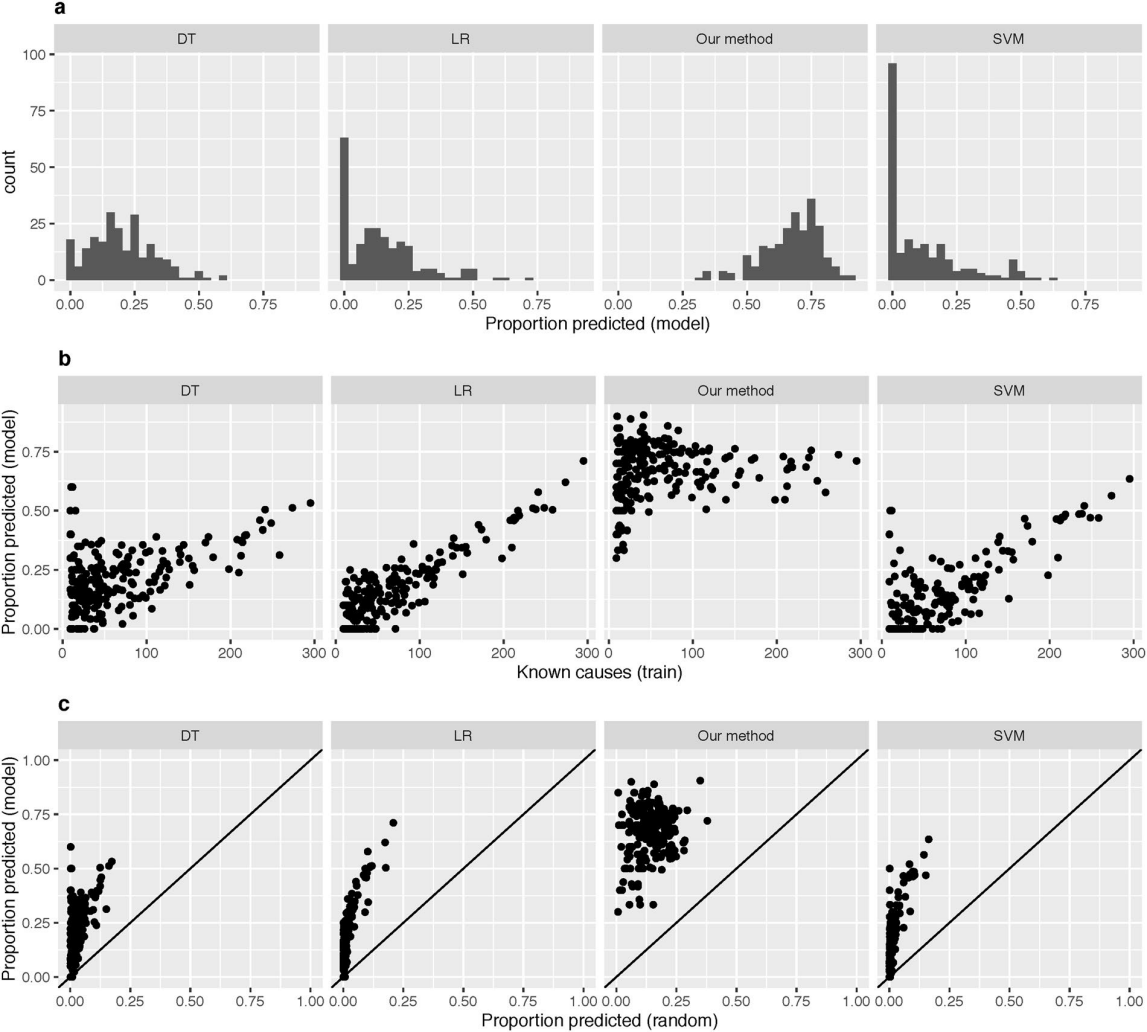
Trained models outperform random and standard models in simulated prediction tasks. (**a**) Distribution of the proportion of deleted edges that was correctly predicted by each method for each ADR, as an average over all folds. (**b**) Average proportion of deleted edges correctly predicted by each algorithm for all ADRs. (**c**) Proportion of deleted edges predicted by trained models compared to the expected proportion achieved by a random model. Solid diagonal line represents identical performance. Points above the line indicate the trained model performed better than random. DT = Decision Trees, LR = Logistic Regression, SVM = Support Vector Machines.

Over all ADRs, 67.3% of deleted edges were correctly predicted by the trained models. As a benchmark, we also tested the performance of several standard machine learning methods that have previously been used for ADR prediction. The benchmark methods used were logistic regression (LR, used for ADR prediction in^11^), decision trees (DT, as used in^13^) and support vector machines (SVM, as used in^19^). Our method gave substantially better performance than all the benchmark methods (67.3% correctly predicted vs. 20.0% for DT, 14.5% for SVM, 14.3% for LR, Fig. 2a). Furthermore, the performance of our method was consistently high independent of the number of known causes of each ADR (Fig. 2b). The performance of all four methods was compared to the expected performance of random guessing (Fig. 2c). The distance of each point from the diagonal (where model performance equals random) was used as a measure of confidence in the ability of the optimised model to outperform a random model in the EHR validation. Our method outperformed random for all ADRs, whereas all other methods were no better than random for a proportion of ADRs (DT 7.9%, SVM 41.9%, LR 27.5%). This large difference in performance is partly due to the high proportion of models that do not make any new predictions for the other methods. Over all folds for all ADRs, our method makes new predictions for 99.7% of models, compared to 92.5% for DT, 52.4% for SVM and 65.1% for LR.

The benchmark methods used here are widely used for classification. We found that our method performed similarly to LR, DT and SVM at classifying the known causes of ADRs (Supplementary Note S1 and Supplementary Figure S2).

### Model validation in Electronic Health Records

The EHR at the South London and Maudsley NHS Foundation Trust was used to validate drug-ADR associations predicted by the algorithm. ADR mentions were identified from the free text of the EHR using a published NLP pipeline developed previously using the same EHR^16^. Reports were only considered a validation of a drug-ADR association when patients were prescribed a single drug and then reported the ADR within 30 days. To evaluate the performance of the prediction algorithm we identified a set of ADRs for validation for which 1) onset would be expected to occur within 30 days, 2) the ADR concept in the knowledge graph can be reliably detected in the EHR text with the NLP pipeline and 3) a predictive model could be built from the knowledge graph. Applying these criteria left a set of 10 ADRs for validation (Table 2). Importantly, as shown in Table 2, these test cases were not selected based on confidence in the predictive model.

**Table 2:** ADRs for which we attempted to validate novel predicted drug associations in the EHR. The “known” column refers to the total number of drugs in the knowledge graph with an edge to each ADR.

Name	UMLS	Known	AUC	High confidence
Akathisia	C0392156	46	0.951	True
Alopecia	C0002170	215	0.857	False
Amenorrhoea	C0002453	62	0.877	True
Galactorrhoea	C0235660	49	0.887	True
Hyperprolactinaemia	C0020514	14	0.973	True
Hypersalivation	C0013132	12	0.982	True
Neuroleptic Malignant Syndrome	C0027849	43	0.965	True
Pericarditis	C0031046	28	0.895	False
Pulmonary embolism	C0034065	67	0.898	False
Stevens-Johnson syndrome	C0038325	165	0.842	False

The predictive performance of each model was first assessed by comparing the number of new predictions made by the trained model that were validated in the EHR to the performance of random models (Table 3). The number of new predictions that were validated in EHR data was greater than expected by chance for all tested models, however for Alopecia and Stevens-Johnson Syndrome a considerable proportion of random models did perform at least as well (14% for Stevens-Johnson syndrome, 36% for alopecia). The confidence grouping derived from cross validation performance compared to random models performed well overall in identifying models that are outperformed by random in <5% of cases in EHR validation (Table 3). The only exceptions were for neuroleptic malignant syndrome and pericarditis.

**Table 3:** Validation of trained models in EHR data. N = number of drugs predicted to cause the ADR that were tested in the EHR data. V = number of predicted drugs that were associated with the ADR (validated) in EHR data. E = expected number of validated predictions given N and the proportion of all drugs that are associated in the EHR. Random models generate N predictions for each ADR, and the trained model is considered significant if <5% of 100,000 random models had an equal or greater validation rate.

Name	N	V	E	Proportion random ≥ trained	Significant	High confidence
Akathisia	22	9	5	0.0337	True	True
Alopecia	18	5	4	0.3556	False	False
Amenorrhoea	20	6	3	0.0386	True	True
Galactorrhoea	22	5	2	0.0329	True	True
Hyperprolactinaemia	14	12	6	6.00E-04	True	True
Hypersalivation	20	15	9	8.00E-04	True	True
Neuroleptic Malignant Syndrome	20	6	3	0.0612	False	True
Pericarditis	19	11	3	1.00E-04	True	False
Pulmonaryembolism	22	8	5	0.1015	False	False
Stevens-Johnson syndrome	22	4	2	0.1434	False	False

### Comparison to existing methods

As a benchmark, we applied machine learning methods that have previously been successfully applied to the ADR prediction task for which well documented implementations are readily available. The predictions generated by previously published methods will depend on the input features, so these methods were trained on the same input features as used with our method, and their prediction performance was evaluated with the same EHR pipeline. In all cases, new predictions were taken as the false positives from the model and the validation rate was compared to random chance.

The benchmark methods are the same as those used earlier in the simulated prediction task: LR, used for ADR prediction in^11^, DT, as used in^13^ and SVM, as used in^19^. The results for all methods are shown in Table 4.

**Table 4:** Prediction performance compared to other methods. By definition the method developed in this paper makes predictions for all 10 of the validation ADRs. The average percent of random models with better performance is calculated considering only the ADRs with at least one validated prediction. LR = logistic regression, DT = decision trees, SVM = support vector machines.

Method	Percent of all ADRs with new predictions	Validation ADRs with new predictions	ADRs with ≥1 predictions validated	Trained model outperforms >95% of random models	Average % of random models with better performance
This paper	91.1	(10/10)	10/10	6/10 (60%)	7.7
LR	52.8	7/10	6/7	1/7 (14%)	24.6
DT	89.7	10/10	4/10	1/10 (10%)	24.8
SVM	46.7	4/10	4/4	1/4 (25%)	36.7

The 10 ADRs used for validation were partly selected because the predictive model made new predictions that could be tested, so all 10 have new predictions, however our method also generated new predictions for more ADRs overall. There was only one ADR for each of the three alternative methods that performed better than randomly selecting the same number of drugs at least 95% of the time (galactorrhoea for SVM and LR, pericarditis for DT). On average over all validation ADRs with new predictions, our method outperformed random 92.3% of the time, compared to 75.4% for the next best method (LR). Therefore, the method presented here both produces new predictions for more ADRs, and the validation rate of new predictions is much better.

### Examples of validated ADR predictions

The overall top 10 highest-scoring predicted ADRs that were validated in EHR data are shown in Table 5. As a secondary validation of these predictions, the European Medicines Agency EurdaVigilance database of spontaneous ADRs was queried for reports of the same association (Table 5). An advantage of the prediction method used here is that the enriched features may provide a molecular mechanism for the ADR^17^.

**Table 5:** The ten highest-scoring predicted ADRs that were not present in the drug knowledge graph and were validated in EHRs. The number of reports of each drug-ADR pair (“Drug + ADR”) and the total number of reports of all ADRs for each drug (“Drug (all)”) are shown for both the EHR used for validation and the EudraVigilance database. The total ADR reports for each drug in the EHR only includes the 10 ADRs used for validation. The EudraVigilance reports include all cases for all ADRs reported in the dataset up to August 2017 (accessed October 2017). Note that the ratio of “Drug + ADR” to “Drug (all)” is expected to be much larger in the EHR as only 10 ADRs are considered, vs all ADRs for EudraVigilance.

Drug	ADR	EHR		EudraVigilance	
		Drug + ADR	Drug (all)	Drug + ADR	Drug (all)
Imipramine	Akathisia	2	4	5	1,465
Trimipramine	Akathisia	1	2	3	931
Amitriptyline	Akathisia	2	13	16	8,832
Quetiapine	Alopecia	18	20	74	34,010
Mirtazapine	Neuroleptic Malignant Syndrome	2	81	81	10,215
Clomipramine	Pulmonary Embolism	1	8	15	3,676
Lamotrigine	Pulmonary Embolism	8	63	29	21,168
Donepezil	Pulmonary Embolism	7	12	15	5,129
Haloperidol	Pulmonary Embolism	6	53	99	9,532
Aripiprazole	Stevens-Johnson Syndrome	3	564	29	17,758

Of the top 10 predictions, 3 are for akathisia and 4 are for pulmonary embolism. Akathisia is a movement disorder and extrapyramidal side effect characterised by a feeling of restlessness and a compulsion to move. A pulmonary embolism is a blockage of the pulmonary artery, which supplies blood to the lungs. An embolism elsewhere can cause pulmonary embolism if the clot dislodges and reaches the lung.

All three drugs associated with akathisia in Table 5 are tricyclic antidepressants (TCAs), and the indications “depression” and “major depression” were predictors in the model. Extrapyramidal side effects, including akathisia, have been associated with TCAs in case reports^20,21^, as well as with the related class of selective serotonin reuptake inhibitor (SSRI) drugs^22^. Extrapyramidal side effects are listed as possible rare ADRs in the data sheets for imipramine and amitriptyline. As noted by Vandel *et al*. in their review of these case reports^20^, given the widespread prescription of TCAs, the incidence of extrapyramidal side effects must be very low to have resulted in only a small number of reports. The underlying mechanism of akathisia remains unclear but is thought to involve dysregulation (hypo-activity) of dopaminergic neurotransmission^23^, which can result from serotonin potentiation by TCAs or SSRIs. This theory is consistent with the predictive protein targets used in the model, which include several serotonin and dopamine receptor subtypes.

The four drugs in Table 5 associated with pulmonary embolism are unrelated to each other in their primary action: clomipramine (TCA), lamotrigine (anticonvulsant), donepezil (cholinesterase inhibitor), haloperidol (antipsychotic). Of these, haloperidol is associated with venous thromboembolism in the knowledge graph, and pulmonary embolism is associated generally with antipsychotics as a class of drugs. One case report was found in which thrombosis occurred following clomipramine treatment^24^. The prediction that these drugs can cause pulmonary embolism was largely based on their other known ADRs. Examples of predictive ADRs in the model with direct relevance to pulmonary embolism include deep vein thrombosis, venous embolism, thrombocytosis, thrombophlebitis and increased prothrombin levels.

## Discussion

At the time a drug is approved for use, only a subset of the possible adverse reactions to that drug will be known from clinical trials. Electronic health records are a vast repository of actual patient outcomes, however much of this data is only contained in the free text. In this paper, we have developed a prediction algorithm that uses publicly available data on drugs, which would be available at the time of marketing, that can predict ADRs observed in health records that are not found in public databases. With this algorithm, we demonstrate a pharmacovigilance pipeline using drug-ADR associations extracted from the free text of an EHR to verify predictions made using a knowledge graph of publicly available data.

A significant distinction between the existing approaches to ADR prediction is whether the predictions are generated for drug-like molecules currently in development, or for drugs that have undergone clinical trials. From a modelling perspective, the key difference between these two situations is the availability of a side effect profile for the drug (albeit a likely incomplete one). Intrinsic structural properties of the drug-ADR network alone can achieve surprisingly high performance in predicting additional ADRs, but the integration of additional data improves performance^11^. ADR predictions for lead molecules have tended to focus on chemical features (such as widely-used quantitative structure-activity relationship models^25,26^), possibly also including gene expression profiles^27^ or drug targets^28,29^. This study is focused on predicting additional ADRs for drugs in the post-marketing phase. In general the method presented could be used to make predictions for lead molecules, but the drug knowledge graph used in this paper is limited to targets, indications and ADRs. The lack of any chemical features means there would be very little input data remaining in the present knowledge graph for a lead molecule.

Integrating chemical features into the knowledge graph could also improve the predictions for marketed drugs, as well as allowing predictions to be made for lead drugs. For example, certain ADRs are related to specific chemical subgroups in the drug molecule^17,30,31^. One way to achieve this integration would be to represent the presence of different chemical substructures as facts in the graph, which have previously been used to predict side effect profiles^30^. Alternatively, Shao *et al*. showed that representing the molecular structure of drugs as a graph and then applying pattern mining techniques to identify features outperformed more widely used methods such as molecular fingerprints for ADR prediction^31^. Drug structure graphs (or features of these graphs) could be represented within the knowledge graph and used to generate predictions, possibly increasing predictive value. Cami *et al*.^11^ used 16 molecular features of drugs as covariates in their ADR prediction model, several of which are continuous values (e.g. molecular weight, rotatable bond count) rather than binary facts used here. An expanded version of the knowledge graph could incorporate these continuous features in the graph as edge weights. Including chemical features would change the input data, so the performance would need to be re-evaluated.

Previous studies^18,27^ found that including GO annotation of target proteins or differentially expressed genes improved classifier performance in the ADR prediction task. The combined GO annotation and ontology forms a knowledge graph, and as such this data could straightforwardly be integrated in the drug knowledge graph constructed in this study. It is therefore possible that expanding the input knowledge graph (for example with chemical features of drugs) would improve the accuracy of the predicted ADRs.

Verifying predicted ADRs using EHRs rather than relying on spontaneous reports has several advantages, the most significant being that this approach overcomes the under-reporting issues of spontaneous report databases. However, there are some general limitations. The most important limitation is that we have to assume patients are complying with their prescriptions, and also that they are not taking any medications not captured in the EHR (which could be the true cause of the ADR). This is particularly problematic for outpatients. As with spontaneous reports, these associations alone do not prove a causal link between a drug and an ADR and we cannot truly establish (and report) a causal link without further manual investigation to rule out other possible causes. To mitigate these limitations, we focused on patients who were only prescribed a single drug and then reported the ADR within 30 days. This increases our confidence that the prescribed drug is associated with the ADR, however it is not a perfect solution. Some ADRs can have chronic onset (such as amenorrhoea, galactorrhoea, alopecia) or may be reported “out of sync” with drug prescriptions, i.e. an ADR could have been caused by a previous medication that was stopped, but the ADR was only recorded after another prescription was given. Considering these limitations, we consider the drug-ADR associations presented here as strong candidates that require further clinical validation.

Considering only those patients prescribed a single drug also excludes a significant proportion of patients who are prescribed multiple drugs concomitantly. Technically the prediction algorithm could straightforwardly extend to predict ADRs for combinations of drugs, increasing its utility in likely real-world contexts where many patients take multiple medications. Including drug combinations is practically challenging as the size of the prediction problem would increase exponentially. Finally, the validation is dependent on reliable NLP to extract ADR mentions from the free text of EHRs. As the pipeline used for validation was developed and validated using the same EHR^16^, we are confident that the error rate is low.

The predictive models generated using our method are essentially sets of drug properties for each type of information in the knowledge graph, along with a weight for each type. The basis for a given drug-ADR prediction is therefore very clear, and the sets of predictors – particularly the predictive targets – may provide valuable mechanistic information for future drug development. As new drugs are developed and added to the knowledge graph, the model optimisation process should be repeated. Although it is possible to use the previous model to calculate a score for a new drug and any ADR, adding edges to the graph would affect all the underlying enrichment calculations that are used to identify predictors. This is computationally expensive and may also result in previous predictions changing. However, this is also a reasonable feature by analogy to the human reasoning process – when we learn new information, it may require us to revise previous predictions. Beyond the task of ADR prediction, the algorithm used here is a general-purpose method that can be applied to any knowledge graph. Further work is needed determine its performance in other contexts.

Machine learning algorithms could become valuable tools for pharmacovigilance, which is a critical element of drug safety. Systems pharmacology methods such as that presented here could be used to predict and understand ADRs that were not observed in clinical trials but are possible given the observed ADR profile and other known properties of the drug. These predictions may not warrant inclusion in drug safety leaflets for patients, but could be provided to clinicians to promote reporting of these predicted ADRs. Looking further ahead, we can envisage a learning healthcare system in which ADRs are automatically detected in patient records and are reported to relevant regulatory bodies if the specific drug-ADR association is missing from the system’s knowledge graph. Such a system lowers the burden on clinicians’ time and mitigates the under-reporting problems of current post-marketing surveillance, potentially improving patient safety. These reports could be used to dynamically generate ever more accurate ADR predictions.

## Methods

### Knowledge graph construction

The knowledge graph was constructed as a Neo4j 3.0 database containing publicly available data on marketed drugs. Data on drug targets was retrieved from DrugBank version 4.5.0 (www.drugbank.ca)^32^. Adverse drug reactions and indications extracted from drug package inserts were retrieved from SIDER (www.sideeffects.embl.de)^33^ on 09/06/2016. Drugs were matched between datasets using PubChem compound identifiers. Target proteins were identified using Uniprot identifiers, indications and adverse drug reactions were identified using Unified Medical Language System (UMLS) terms. The constructed graph contains 4 types of node (drug, ADR, indication, target) and 3 types of edge representing each relationship. Every edge in the graph has a drug node at exactly one end. Only drugs with at least one edge of each type were retained in the final knowledge graph (i.e. each drug must have at least one known target, indication and ADR). The complete public knowledge graph used in this study is provided in Supplementary Table S1.

### Prediction algorithm

The prediction algorithm is a binary classifier which is used to place edges in a graph. For each ADR node, the knowledge graph is queried to find known causes. For each of the three types of drug knowledge in the graph (target, indication and ADR), Fisher’s exact test is used to identify enriched properties of the known causes of the ADR vs. all other drugs in the graph (excluding the ADR being modelled). Enriched properties are those with a p-value for enrichment <0.05 following false discovery rate correction. These identified properties are nodes in the knowledge graph, and the raw features used to make predictions are the adjacency of all drug nodes in the graph with these (enriched) nodes. Standard feature scaling is applied to each feature type separately to scale values to the range 0–1. Feature scaling is important as drugs typically have many more known ADRs than targets or indications.

The scaled features can be represented with matrix D, where each row corresponds to a drug and each column corresponds to a predictor node type. The value D_i,j_ is therefore the total (scaled) adjacency of drug i with all predictors of type j. The score for each drug is calculated by multiplying D by a weight vector w (to be learnt from the data), which contains the weight of each predictor type in the same order as the columns of D. A drug is predicted to cause the ADR if its score is greater than a threshold. New predictions from the model are any drugs that are not known causes of the ADR that score higher than the threshold, and there may not be any new predictions for a given model.

The feature weights and score threshold are selected to maximise the objective function. In general, any objective function could be used to determine the threshold, based on the cost context of false positives vs false negatives, and the weights for each predictor type could be varied over any given range. For this study, the weights for all predictor types were kept within the range 0–1 with L2 normalisation. Youden’s J statistic was used as the objective function, which is defined as J = Sensitivity + Specificity − 1 (equation 1). Sensitivity is the true positive rate, meaning the proportion of all positive examples that was correctly identified. Specificity is the true negative rate, meaning the proportion of all negative examples that was correctly identified.

1 $$J=\,\frac{TP}{P}+\,\frac{TN}{N}-1=Sensitivity+Specificity-1$$

At least one predictor for each node type was required before training a model. This requirement for predictors of all types to be identified meant that it was not possible to build a model for all ADRs in the training data. Relaxing this constraint would allow a greater number of predictive models to be built.

The prediction algorithm is implemented as an open source python library which available and documented in detail at https://github.com/KHP-Informatics/ADR-graph.

### Benchmark methods

Machine learning methods that have previously been successfully applied to the ADR prediction task and for which well documented implementations are readily available were tested used as benchmarks. The selected methods were logistic regression (LR), support vector machines (SVM) and decision trees (DT), all of which are implemented in scikit-learn^34^ version 0.17.1. SVM classifiers used the RBF kernel and default settings to emulate the SVM classifiers used in^19^. DTs were configured to emulate the method of Bresso *et al*.^13^ by setting the minimum samples per leaf to 5. LR classifiers used L2 regularisation and default settings. All benchmark machine learning methods were trained on the same input data as described for our method (normalised adjacency with enriched properties of known causes of each ADR). False positives from all models were taken as new predictions and validated using the same EHR pipeline.

### Random models

For each ADR, 100,000 random models were generated that made the same number of new predictions as the trained model for the same ADR. Random models selected drugs uniformly at random from the list of all drugs that are not known to cause the ADR and are prescribed in the EHR used for validation. The predictions from these random models are validated in the EHR using the same pipeline as for the trained models. The proportion of random models with at least as many validated predictions as the trained model was used as the performance metric for trained models (Supplementary Figure S3). Trained models with more validated predictions than >95% of random models selecting the same number of drugs were considered significant.

### Cross-validation

To simulate the prediction task, the edges for each ADR node were divided into 10 folds. In each fold, 1/10^th^ of the edges to drugs from an ADR were deleted and a model was then optimised using the resulting graph. The proportion of deleted edges that are correctly predicted by the model is determined. Importantly, only edges (not nodes) are deleted from the graph in each fold. This means the drug nodes that were previously connected to the ADR remain in the graph and are considered true negatives when the model is trained. This is a more accurate simulation of the intended prediction task than a standard cross-validation where the test set is completely held out.

Cross validation performance was used to derive a confidence score for the predictions for each ADR generated on the full graph. The raw confidence is average proportion of deleted edges that was correctly predicted over all folds, relative to the expected performance of a random model. If the relative performance equals 1, the model performs as expected by random chance. These raw scores were binned into confidence groups, where high confidence models score better than the median and mid confidence models score better than random but less than the median. Any ADRs with performance less than or equal to random are assigned low confidence. ADRs with fewer than 6 successful folds were not analysed, and were assigned low confidence by default.

### Validation in electronic health records and Eudravigilance

De-identified patient records were accessed through the Clinical Record Interactive Search (CRIS)^35^ at the Maudsley NIHR Biomedical Research Centre, South London and Maudsley NHS Foundation Trust. This is a widely used clinical database with a robust data governance structure which has received ethical approval for secondary analysis (Oxford REC C 08/H0606/71 + 5). Free text from these mental health electronic health records was processed using a Natural Language Processing pipeline described previously^16^. Briefly, ADR mentions are identified using a dictionary of related terms (including synonyms and misspellings) and further processed to identify negation or other experiencers, favouring precision over recall. In this pipeline, ADRs are any adverse events in the record that could be an ADR, although causality is not established^16^.

When an ADR mention is detected in the free text, we associate it with all drugs prescribed within the previous 30 days based on the typical onset of the ADRs used for validation. Only ADR mentions with a single associated prescription were considered in the validation; reports from patients prescribed more than one drug in the previous 30 days are ignored. The Eudravigilence database was queried via www.adrreports.eu on 04/10/2017. All ADR reports up to August 2017 were retrieved.

### Data availability

The data on drug indications, ADRs and targets are publicly available (see “Knowledge graph construction”) and the knowledge graph produced from this data is available as Supplementary Table S1. The prediction algorithm is implemented as an open source python library, available and documented with the knowledge graph at https://github.com/KHP-Informatics/ADR-graph. The anonymised EHR data are available for secondary research via CRIS^35^ subject to approval by the CRIS Oversight Committee in adherence with strict patient-led governance^35^.

## Electronic supplementary material

Supplementary information

Supplementary Table S1
