# Author Correction: Knowledge graph prediction of unknown adverse drug reactions and validation in electronic health records

**DOI:** 10.1038/s41598-018-22521-4

**Published:** 2018-03-06

**Authors:** Daniel M. Bean, Honghan Wu, Ehtesham Iqbal, Olubanke Dzahini, Zina M. Ibrahim, Matthew Broadbent, Robert Stewart, Richard J. B. Dobson

**Affiliations:** 1 Department of Biostatistics and Health Informatics, Institute of Psychiatry Psychology and Neuroscience, King’s College London, 16 De Crespigny Park, London, SE5 8AF United Kingdom; 2 South London and Maudsley NHS Foundation Trust, Denmark Hill, London, SE5 8AZ United Kingdom; 3 Institute of Pharmaceutical Science, King’s College, London, 5th Floor, Franklin-Wilkins Building, 150 Stamford Street, London, SE1 9NH United Kingdom; 4 Institute of Psychiatry, Psychology and Neuroscience, King’s College London, 16 De Crespigny Park, London, SE5 8AF United Kingdom; 5 Farr Institute of Health Informatics Research, UCL Institute of Health Informatics, University College London, London, WC1E 6BT United Kingdom

Correction to: *Scientific Reports* 10.1038/s41598-017-16674-x, published online 27 November 2017

Ehtesham Iqbal and Zina M Ibrahim were omitted from the author list in the original version of this Article. This has been corrected in the PDF and HTML versions of the Article, and the accompanying Supplementary Information.

The Author Contributions section now reads:

D.B., H.W. and R.J.B.D. designed the study. O.D. advised on validation. H.W. and E.I. processed EHR data. D.B. designed the prediction algorithm, performed analysis and prepared the manuscript. R.J.B.D., Z.M.I., M.B. and R.S. supervised the project. All authors reviewed the manuscript.

In addition, the original version of this Article contained an error in Reference 16.

“Iqbal, E. *et al*. Identification of Adverse Drug Events from Free Text Electronic Patient Records and Information in a Large Mental Health Case Register. *PLoS One* 10, 1–14 (2015).”

now reads:

“Iqbal, E. *et al*. ADEPt, a semantically-enriched pipeline for extracting adverse drug events from free-text electronic health records. *PLoS One* 12, 1–16 (2017).”

