# Supplementary material for: Knowledge graph prediction of unknown adverse drug reactions and validation in electronic health records
Source: Sci Rep. 2017 Nov 27;7:16416. doi: 10.1038/s41598-017-16674-x (PMC5703951; doi:10.1038/s41598-017-16674-x)
Supplement: Supplementary file 1 — Supplementary information [file 41598_2017_16674_MOESM1_ESM.docx]

Knowledge graph prediction of unknown adverse drug reactions and validation in electronic health records

Daniel M. Bean^1^, Honghan Wu^1^, Ehtesham Iqbal^1^, Olubanke Dzahini^2,3^, Zina M. Ibrahim^1,5^, Matthew Broadbent^2^, Robert Stewart^2,4^, Richard J. B. Dobson^1,5*^

1. Department of Biostatistics and Health Informatics, Institute of Psychiatry Psychology and Neuroscience, King’s College London, 16 De Crespigny Park, London, SE5 8AF, United Kingdom

2. South London and Maudsley NHS Foundation Trust, Denmark Hill, London, SE5 8AZ, United Kingdom

3. Institute of Pharmaceutical Science, King’s College, London, 5th Floor, Franklin-Wilkins Building, 150 Stamford Street, London, SE1 9NH, United Kingdom

4. Institute of Psychiatry, Psychology and Neuroscience, King’s College London, 16 De Crespigny Park, London, SE5 8AF, United Kingdom

5. Farr Institute of Health Informatics Research, UCL Institute of Health Informatics, University College London, London, WC1E 6BT, United Kingdom

Contact

RJBD*: richard.j.dobson@kcl.ac.uk

*Corresponding author

**Supplementary information**

Figures S1, S2, S3

Table S1 (separate file)

Note S1

**Supplementary figures**

**Figure S1**. Distribution of drug-ADR edges in the knowledge graph. A) Distribution of the number of known ADRs for each drug node. B) Distribution of the number of known causes (drugs) for each ADR node.

**Figure S2**. Distribution of AUC over all ADRs. There are 356 ADRs with predictive models. All methods were trained on the same features. DT = Decision Trees, LR = Logistic Regression, SVM = Support Vector Machines.

**Figure S3.** Generation of random benchmark models. 100,000 random models are generated for each ADR Y. For each model, d drugs are selected from the list of all drugs that are not currently known to cause Y and are prescribed in the EHR, where d is the number of predictions made by the (single) trained model for Y. The performance of all models (random and trained) is measured by the proportion of new predictions that are validated in the EHR. By chance, some of the random models may perform better than the trained model (dashed line, right). This provides a measure of how likely it is to observe performance at least as good as that of the trained model by chance alone.

**Supplementary tables**

Supplementary Table S1 is provided as a separate file.

Supplementary Table S1. Edges in the final drug knowledge graph. Only drugs with at least one edge of each type (drug-ADR, drug-indication, drug-target) are included. Drugs are represented by DrugBank identifier (www.drugbank.ca), proteins by UniProt identifier (www.uniprot.org), ADRs and indications by Unified Medical Language System (UMLS) concept identifier (https://www.nlm.nih.gov/research/umls/).

**Supplementary notes**

**Supplementary Note S1. Classifying known causes of each ADR**

The predictive models are based on using an enrichment test to identify edges that are more likely to exist for the known causes of an ADR than for all other drugs. The enrichment test is applied separately for each type of node in the graph. For most ADRs, there is at least one type of drug knowledge for which there are no enriched properties. To study the performance of the prediction algorithm under consistent conditions across ADRs, we focused on only those ADRs for which predictors were identified for all node types. With these conditions, applying the prediction method to all ADRs in the graph resulted in 356 optimised predictive models.

Using these trained models for each ADR, the area under the receiver operator characteristic curve (AUC) was calculated as a measure of the classification performance of a model with these weights independently of the threshold. As a benchmark, we compared our method to the performance of DT, SVM and LR classifiers trained on the same data. The AUC distribution over all ADR models is shown in Figure S2. When classifying known causes of an ADR, our method had essentially identical performance to LR (average AUC 0.919 for our method vs 0.918 for LR). Both LR and our method were slightly worse than DT (average AUC 0.967) and all methods were better than SVM (average AUC 0.88). Our method has excellent classifier performance on average, and even the worst performing model had an AUC of 0.77. Therefore the weighted features are representative of the properties of drugs that are known to cause a given ADR, and may be useful to identify new (unknown) causes.

Given that the optimised weights produce classifiers with very good performance, the weights for each predictor type are informative. Overall, other ADRs of the drugs known to cause the ADR being predicted were the most used feature (weight > 0 in 96% of models), followed by indication (67%) and targets (57%). 42% of models used all 3 predictor types.

The present study is focused on ADR prediction, but the method developed here is not specific to ADR prediction. As an example, we also trained models using the same method and knowledge graph to classify target proteins and indications for all drugs. The indication classifier achieved an average AUC of 0.978 for the 141 indication nodes with models. For drug targets, the average AUC was 0.982 for 77 targets with models. Therefore the use of enriched properties of the known associated drugs as input features produces very good classifier performance in different contexts.
